# Analysis of Technical–Tactical Actions in High-Level Table Tennis Players: Differences between Sexes

**DOI:** 10.3390/sports11110225

**Published:** 2023-11-14

**Authors:** Francisco Pradas de la Fuente, Miguel Ángel Ortega-Zayas, Víctor Toro-Román, Alejandro Moreno-Azze

**Affiliations:** 1 ENFYRED Research Group, Faculty of Health and Sports Sciences, University of Zaragoza, 22001 Huesca, Spain; franprad@unizar.es (F.P.d.l.F.); maortega@unizar.es (M.Á.O.-Z.); amazze@unizar.es (A.M.-A.); 2 School of Sport Sciences, University of Extremadura, 10003 Cáceres, Spain

**Keywords:** racket sports, motor skills, stroke, winner, loser, male, female

## Abstract

Table tennis is a sport played at a high speed; therefore, the technical–tactical variables are very important. The objective of the research is to analyze the technical and tactical characteristics of high-level TT players according to sex. A total of 48 high-level players (24 women and 24 men) participated in the present study. The investigation was carried out during two championships. The matches were recorded and subsequently analyzed by notational analysis. The results indicate that women stroke the ball more times during the rallies. In the men’s competition, the forehand technique predominates over the backhand technique. The flip was the most used in the male sex (*p* < 0.05). At the tactical level, more winning actions were performed in the men’s competition than in the women’s, both with the forehand and backhand game. Men performed more losing technical actions when using the forehand and backhand flips. The pivot footwork tactical action was higher in the men’s competition. The analysis of the technical–tactical actions highlighted important differences between the sexes. The predominant losing techniques among players are forehand and backhand flip. Female players use more defensive strokes, while male players use more offensive strokes, in particular the flip technique. The potential biomechanical progress of the male player characterized by a larger wingspan biotype could facilitate a better technical–tactical performance. The results obtained are of interest to improve the performance of the players as they must train at a technical–tactical level differently depending on the sex and style of play.

## 1. Introduction

Table tennis (TT) is a sport played by approximately 300 million people worldwide, of which at least 40 million are federated players [1]. TT is a racket sport characterized by fast, intermittent, and high-intensity playing actions [2]. TT is considered a sport with great structural complexity as it requires a wide range of technically different strokes, which, among other factors, depend on the material used in the racket and the style of play [3].

TT players stroke the ball more than 30 times per minute during games with a maximum duration of 4 s and with rest times of less than 15 s. The duration of games differs between sexes, ranging from 8 to 38 min among male players and from 9 to 41 min among female players [4].

Considering the characteristics and game dynamics described for this sport, TT should be considered one of the fastest sports in terms of speed of play [5]. High-level players present high perceptual–motor skills and automated and anticipatory patterns of play [6]. In addition, they master superior tactical skills and sustain physical demands [7].

TT’s technical and tactical actions require functional pairing between perceptual and action modalities under different spatial and temporal demands [8]. The competition of TT includes complex motor skill learning, numerous repetition, and multisensory feedback, and all these features can foster the emergence of neural plasticity and executive functioning [9].

To perform a stroke during a match, the TT player must pay full attention to the stimuli (visual and auditory) that appear in each game action, to gather all the necessary information at the cerebral level to indicate to the neuromuscular system the most appropriate response depending on the circumstances of the game [10]. The athlete must then make multiple neuromuscular decisions involving physical actions (movements), techniques (selection of the type of stroke to perform), tactics (delivery zone of the ball), and biomechanics (acceleration of the gesture to be performed, angle of impact, and racket-ball contact time) [11]. Regarding the above, previous authors observed a significant difference in topspin stroke between elite and medium athletes in the forward and complete phases. In addition, elite athletes had a total hip and knee movement in the complete phase and a better lower-extremity-driving ability during the topspin forehand loop compared to medium athletes [12]. All this process is carried out in a period that can last for less than a second in certain situations.

Multiple factors influence TT competition, such as mental abilities, anthropometric characteristics, physical fitness, technique, and tactics, with the latter two factors having a significant impact [13,14]. The technical and tactical analysis of TT competitions can provide a helpful training guidance and improve players’ competitive ability [15]. Therefore, the analysis of the technical and tactical characteristics of TT players seems to be a key aspect in their preparation to reach optimal performance levels [16].

While team sports have attracted a relatively large number of research regarding the analysis of the technical–tactical elements that occur during their play, there are very few studies on this topic focusing on TT [3]. Previously, instruments to evaluate technical–tactical actions during TT play have been reported [17,18,19], as well as the analysis of the different technical–tactical actions of players in matches [3,20,21]. However, the studies carried out to date are very basic, limited, and heterogeneous because they study very small samples and of different performance levels or analyze a very small number of matches, with the participants being mostly men. Given the above, this study aims to analyze the technical–tactical characteristics of high-level TT players and to differentiate these actions between the sexes.

## 2. Materials and Methods

### 2.1. Subjects

The sample selection for this research was carried out by employing non-probabilistic purposive sampling. A total of 48 high-level TT players, 24 males and 24 females of different nationalities, were selected for the research. The nationalities of the players were: male (10 Spanish; 6 Chinese; 1 Austrian; 2 Hungarian; 1 Nigerian; 2 Ukrainian; 1 Indian; and 1 Russian) and female (10 Spanish; 7 Chinese; 2 Dutch; 2 Rumanian; 1 Belarusian; 1 Russian; and 1 Serbian) (Table 1). The technical characteristics of the participants are shown in Table 2.

TT players met the following criteria: (a) senior category (≥18 years old); (b) be federated by the Royal Spanish Table Tennis Federation; (c) play in the top-level league competition of the Royal Spanish Table Tennis Federation; (d) minimum sporting experience of 10 years; and (e) be ranked in the male or female ranking of the senior category of the Royal Spanish Table Tennis Federation in a position no lower than 30th.

The Clinical Research Ethics Committee of the Department of Health and Consumption of the Government of Aragon approved this research (19/2010). All participants were informed of the procedures to be followed during the research and signed a consent form.

### 2.2. Study Design

The present non-experimental, cross-sectional, observational, and descriptive study was carried out in Seville (Spain) during the development of two competitions, the Spanish Absolute Championship, from the round of 16, and the final phase of the Spanish International Open. A total of 24 matches (12 men’s and 12 women’s matches) were observed to carry out the research. Specifically, 1177 plays and 5319 strokes were analyzed in the men’s competition, while 950 plays and 5097 strokes were analyzed in the women’s competition.

### 2.3. Analysis of Technical–Tactical Actions

The fundamental types of strokes considered in this research to carry out the technical–tactical analysis were the following [10,22]: 

#### 2.3.1. Offensive Techniques

(a) Drive: an interlocutory stroke imparting no effect on the ball.
(b) Topspin: an attacking stroke imparting a topspin effect to the ball.
(c) Flip: an attacking stroke performed when the ball bounces close to the net.
(d) Smash: an attacking stroke characterized by a linear trajectory and no ball spin.
(e) Drop, shot when the opponent is far from the table.

#### 2.3.2. Defensive Techniques

(a) Push: an interlocutory stroke imparting a backspin effect to the ball.
(b) Chop: In an extreme backspin shot, the ball travels in a flat trajectory and bounces low.
(c) Block: a defensive stroke performed passively in response to a top.
(d) Lob: a defensive stroke performed when the player is far from the table, lifting the ball to a considerable height.

**Figure 1:**
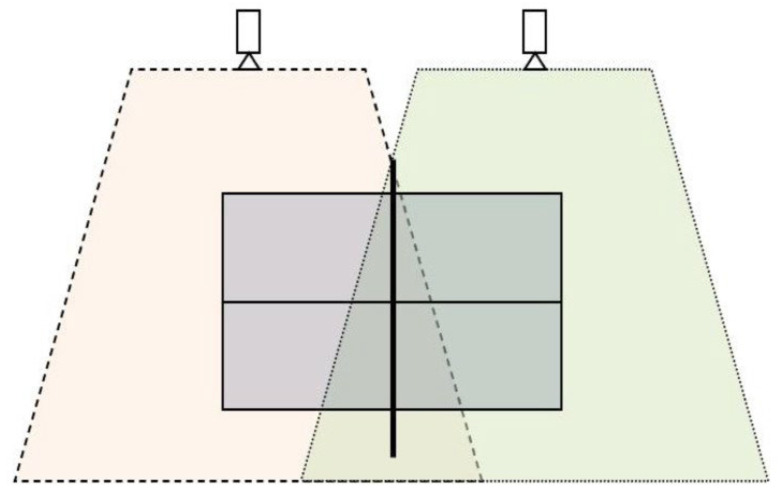
Competition recording protocol [4].

The selected matches were recorded with two Sony HDR-CX300E video cameras (Sony, Japan) to analyze all the technical–tactical actions performed by the players. The cameras were strategically placed to one side and parallel to the game table. Both cameras were positioned 3 m from the competition and elevated from the ground at a height of 2.5 m (Manfrotto, 007BU, Cassola, Italy). All matches were recorded with a shutter speed of 1/500 s. Each camera recorded one side of the game table (Figure 1).

**Figure 2:**
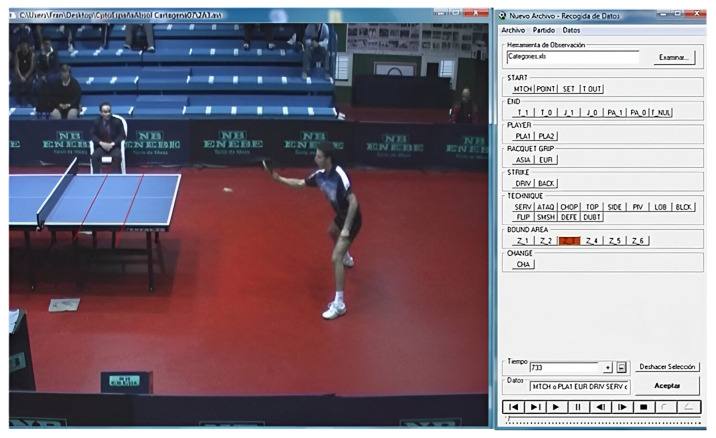
Analysis of the technique with Match Vision Studio^©^ [17].

Then, a synchronization process of both cameras was performed to analyze the sequence of each technical game action. All recorded matches were analyzed by means of a validated observation tool [17], using the Match Vision Studio^©^ v. 3.0 software [23]. The observation tool was organized using an ad hoc annotation system (Figure 2).

Two expert TT coaches with the highest federal qualification of the Royal Spanish Table Tennis Federation (level III) analyzed the matches. The intra- and inter-observer data concordance analysis showed a Kappa index higher than 0.80 in all the analyzed technical and tactical variables, and the obtained degree of agreement was considered to be very high [24].

### 2.4. Statistical Analysis

The statistical analysis was conducted using the IBM^®^ SPSS^®^ Statistics Version 22 program (IBM Corp., Armonk, NY, USA). 

Qualitative or categorical variables were expressed as frequencies and percentages of the different categories. The comparison between both sexes was made using contingency tables using Pearson’s chi-squared. 

Quantitative variables were expressed as the mean and standard deviation (X ± SD), maximum and minimum (range). Normality was assessed using the Kolmogorov–Smirnov test. A Student’s *t*-test was used to compare the means between men and women for normally distributed inter-group variables. In contrast, the non-parametric Mann–Whitney U-test was applied for non-normally distributed variables. For intra-group analyses (right-handed vs. left-handed), a Student’s *t*-test was used to compare related samples, while for variables without a normal distribution, the non-parametric Wilcoxon test was applied to compare the mean rank of two related samples. 

The effect size (ES) was calculated for parametric and nonparametric data [25]. ES values of 0.2, 0.4, and 0.8 were considered small, moderate, and large, respectively [26].

All statistical comparisons were bilateral and a *p*-value of ≦ 0.05 was considered statistically significant.

## 3. Results

The results obtained about the different variables and technical–tactical aspects are presented below. The data are expressed as percentages or mean and standard deviation, sometimes indicating the range (maximum and minimum). 

Firstly, Table 3 shows the results on the number and side of strokes. Female players stroke the ball more times than male players during the games played (*p* < 0.05; ES = 0.82). On the other hand, the intra-group comparisons indicated that male players predominate in the use of the forehand technique over the backhand technique (*p* < 0.01; ES = 1.05).

Table 4 shows the different types of strokes during the matches analyzed. Men stroke more frequently with the flip technique than women (*p* < 0.01; ES = 1.02). Female players made more chop shots than male players (*p* < 0.01; ES = 1.10). In the rest of the strokes, there were no significant differences between sexes.

The results related to the tactical actions developed, in terms of their differentiation considering the side of the game on which the winning and losing game actions occur, are presented in Table 5. Differences between sexes were observed in the winning actions of forehand and backhand (*p* < 0.05; ES = 0.83), being superior in men. Likewise, there were differences in both sexes in the winning forehand and backhand actions, with forehand actions being superior (*p* < 0.05; ES = 0.81).

Table 6 shows the tactical results of the technical winning strokes differentiated by sex. Male players had higher winning strokes in topspin forehand (ES = 0.45), topspin backhand (ES = 0.83), and flip backhand (ES = 0.41) (*p* < 0.05) than female players. On the other hand, there were sex differences in topspin forehand and backhand winners (*p* < 0.05).

Table 7 shows the tactical results obtained regarding the losing technical actions differentiated by sex. The results found indicate a more significant number of errors in the men’s competition in the offensive technique of the forehand and backhand flips (*p* < 0.05; ES = 0.82), as well as the forehand push (*p* < 0.05; ES = 0.42).

On the other hand, there were differences between the forehand and backhand topspin strokes in both sexes (*p* < 0.05), with the forehand stroke being superior. However, in the block and drive strokes, the backhand was more predominant than the forehand (*p* < 0.05).

The results corresponding to the tactical action of the direction of play (diagonal or parallel) and the technical action of the pivot footwork are shown in Table 8. The pivot footwork tactic was superior in males compared to females (*p* < 0.05; ES = 0.51). 

## 4. Discussion

TT is a sport in which control and mastery of technique are crucial. To perform at a high level in this sport, TT players must develop and control their technique in an outstanding manner and with great skill, as well as possessing the ability to have tactical thinking that can be executed instantaneously, due to the speed at which their game is played. Skills such as changing position quickly to adjust the stroke technique, footwork, anticipating and reacting before the shot, proper positioning, and balance control are crucial in this sport [27]. In the last two decades, TT has changed considerably with significant modifications in its rules and game characteristics (size and material of the ball, scoring system, incorporation of time-outs, and changes in the execution of the service) [28]. These modifications have led to an evolution towards a more modern TT, which differs considerably from that played a few years ago regarding the game’s structure and physical demands [29]. The above has led to important game dynamics modifications, forcing coaches and players to adapt to this new sporting scenario.

The technique is the most important training variable in TT. Within a player’s sports planning, depending on the moment of the season, technical work could comprise from 30% to 80% of the total necessary preparation [30]. Strokes and movements are the main technical aspects in this sport, and both are closely linked. To correctly perform a certain technique, it is essential to carry out different movements with the lower body to guarantee a good body position in space concerning the ball, allowing for a greater balance and stability in the stroke [31].

More strokes are made in the men’s competition than in the women’s competition. However, the female players stroke the ball more times than the male players during the rallies played in a game. These results are similar to those described for men [29,32]. However, the results about the female modality cannot be compared as we have yet to find updated reference data in the specialized literature consulted. On the other hand, the game actions carried out using technical executions of the forehand side predominate in both sexes over those carried out with the backhand side [29,32,33], However, female players use both sides of the racket more evenly than male players. Women likely use both sides of the racket to play close to the table against the ball with a backspin. At the same time, men may look for opportunities to stroke topspin, a shot with greater force and spin usually made from the forehand side and somewhat further away from the table [34], using the technical resource of the pivot footwork more frequently than women for this reason. 

The topspin is the type of stroke most frequently used by both sexes in competitions [33]. This technique maintains the initiative in the game, being considered the most aggressive that can be applied to stroke the ball at high speed and with high rotation [35,36]. The flip technique is used to a greater extent in men’s competition. This stroke performed inside the table, also known as a mini-stroke, depends on different perceptual–motor skills, as well as the technical ability to accelerate the racket significantly; this variable is considered a relevant factor that affects performance levels during the game [37], especially in the one performed inside the table. 

The differences found in this type of stroke between sexes could be explained by the biotype, since, on the one hand, a large wingspan facilitates access to short balls. On the other hand, the important muscle masses of the trunk, hip, and shoulder girdle can provide the opportunity to generate a greater force and acceleration to the players. In this sense, Iino et al. [38] highlighted in their research the importance of variables related to the acceleration of the racket, rather than speed, for the impact against balls with a cut effect, as it happens when using the flip technique on serves made to areas close to the net. The ability to accelerate the racket in less time is one of the important factors for the forehand against the slice effect [38], which would corroborate the above and could provide an answer for why, in women’s competitions, there is a greater use of the slice technique, on short balls, while in men’s competitions, the flip technique is more used in this type of game situations. 

Tactic is another of the fundamental aspects of TT, and it is estimated that it can account for between 10% and 60% of the total preparation of a player [30]. It is not easy to describe indicators to define tactics in this sport, and there needs to be a clear and homogeneous consensus to determine, classify, and analyze the tactical actions that characterize it. Thus, some authors have focused on the study of the effectiveness of the strokes, the type of error, the place where the ball bounces on the table, the duration of the phases of play, and the result obtained [39,40,41,42]. On the other hand, several authors distinguished tactics differently and classified them according to the strokes, considering them as winners, transient, and losers [20]. However, other important indicators for analyzing tactics are described in the reference literature, which are based on studying the sequence of strokes, the link between the execution of the service and the following stroke, and the so-called lucky strokes [42]. Given this somewhat indeterminate situation of tactics in TT, it is certainly complex to discuss the data obtained due to the significant diversity among the different studies and research when dealing objectively with tactics in this sport. The winning and losing game actions, and consequently the winning and losing techniques, have in both sexes a direct link with the service made and with the rest [43], as well as with the distance from the table at which it is hit, and with the phase of the game that is being developed, whether defense or attack [3]. On the other hand, more errors are committed in the offensive style of play than in the defensive [43]. In this research, the tactical indicators described were considered individually; therefore, the results obtained cannot be compared since they cannot be related to play sequences. 

The game actions associated with winning and losing plays coincide with the studies carried out, being in both cases and both sexes the topspin technique, considered as a technical action of risk with which more failures occur, and in turn also manifests itself as the most effective in this research, as well as in other works [11]. To our knowledge, there is no current research to compare the tactical indicators corresponding to the direction and pivot footwork, since other researchers have applied notational systems different from the one used in this study [42,44]. In addition, no current research on tactical indicators in the female game has been found. 

From the point of view of physical fitness, the different technical–tactical results found in this study could be explained as a consequence of the differences described in the research carried out in TT, where it is indicated that men are faster in lateral displacement, with considerable lower body strength in its impulsive and elastic–impulsive manifestations, and higher values of isometric strength in the upper limbs [45,46]. Therefore, in men’s competitions, it is possible to develop plays of shorter duration characterized by fast and explosive technical–tactical actions, where offensive shots, such as topspin and flips, predominate. This type of game is facilitated by sexual dimorphism. Generally, men have a greater height and wingspan compared to women [47]. All of the above allows, at a technical level, the development of more powerful strokes, being able to apply a higher speed of rotation and translation on the ball [48,49].

However, specific studies have yet to be found where the technical–tactical response of TT is analyzed in terms of the development of brain processes combined with their differences between sexes. The neurological adaptations between male and female players differ depending on the years of deliberate practice and continuous learning in a specific domain [50,51]. Both processes alter functional brain activity, which is associated with optimal sport behavioral performance and differentiated because of different training designs [51]. 

These results suggest that high motor skills in table tennis are associated with the focused excitability of the motor cortex during reaction and movement planning and execution with high attentional demands [52]; these processes probably differ between men and women. It seems that the practice of TT is of interest in the development and maintenance of brain health in both sexes. 

The limitations of the study are: (i) the sample size; (ii) the style of play developed was not considered; (iii) the playing material used was not considered; and (iv) the dominance of the players was not considered. The stress generated during the competition may affect the technical and tactical actions studied. Future research on the subject should consider the style of play and the material used to homogenize and corroborate the results of the present manuscript.

## 5. Conclusions

The technical and tactical actions used during matches in high-level TT players are different between the sexes.

During the game dynamics of high-level TT players, the most used hitting side by both sexes is the forehand. The total number of strokes in a match is higher in the men’s competition. On the other hand, female players use more defensive strokes, while men use more offensive strokes, particularly the flip technique.

Winning game actions are higher in male players. As for the winning techniques, they are also superior in the male competition, with the predominance of forehand topspin and backhand topspin actions. The players’ losing techniques are also superior, specifically in the forehand flip and backhand flip techniques. The tactical action of the pivot is superior in the men’s competition.

The differences between sexes show different game dynamics between sexes; therefore, there are two TT dynamics, one male and one female. This finding is of interest for coaches and players’ performance, as, during training, this knowledge should be applied at the technical and tactical level differently according to the sex and the playing style. 

## Figures and Tables

**Table 1:** Subjects’ characteristics.

Parameters	Male (n = 24)	Range	Female (n = 24)	Range
Age (years)	25.38 ± 4.00	19.0–38.0	22.30 ± 3.80 *	18.0–31.0
Experience (years)	16.04 ± 4.12	10.0–30.0	13.25 ± 3.85 *	10.0–22.0
Weight (kg)	69.90 ± 9.25	50.8–89.6	57.62 ± 6.20 *	48.3–69.8
Height (m)	1.75 ± 0.06	1.62–1.88	1.65 ± 0.06 *	1.52–1.75
Body mass index	22.60 ± 2.30	18.8–27.2	20.94 ± 1.61	18.3–24.4

* *p* < 0.05 differences male vs. female; values represented in X ± SD.

**Table 2:** Technical game characteristics of the participants.

Parameters (n)	Male (n = 24)	%	Female (n = 24)	%
Right-handed	17	70.8	20	83.3
Left-handed	7	29.2	4	16.7
Defensive style	1	4.2	5	20.8
Offensive style	23	95.8	19	79.2
Shake-hand grip	18	75.0	23	95.8
Pen-hold grip	6	25.0	1	4.2
Pimples-in rubber (R)	21	87.5	23	95.8
Short pimples-out rubber (R)	3	12.5	1	4.2
Pimples-in rubber (B)	22	91.8	18	75.0
Short pimples-out rubber (B)	1	4.1	1	4.1
Long pimples-out rubber (B)	1	4.1	5	20.9

R: forehand; B: backhand; values represented in frequencies and percentages.

**Table 3:** Types of stroke.

Parameters (n)	Male (n = 24)	Range	Female (n = 24)	Range
Total strokes	440.58 ± 147.10	225.0–649.0	424.83 ± 158.84 ^µ^	232.0–795.0
Rally strokes	4.37 ± 0.42	3.8–4.9	5.21 ± 1.12 * &	3.7–7.4
Minimum	1.08 ± 0.28	1.0–2.0	1.33 ± 0.49	1.0–2.0
Maximum	15.08 ± 4.46	10.0–27.0	16.25 ± 5.46 ^µ^	10.0–28.0
Forehand	290.08 ± 106.21	144.0–473.0	227.92 ± 117.01 ^µ^	101.0–535.0
Backhand	153.00 ± 57.62 ++ &	80.0–259.0	196.83 ± 72.50	122.0–356.0

* *p* < 0.05 differences male vs. female; ++ *p* < 0.01 differences forehand vs. backhand; ^µ^: Non-parametric Mann–Whitney U-test; &: large effect size; values represented in X ± SD.

**Table 4:** Stroke techniques.

Parameters (n)	Male (n = 24)	Range	Female (n = 24)	Range
Service	99.67 ± 29.06	59.0–138.0	80.25 ± 18.30 $	60.0–109.0
Drop	0.17 ± 0.58	0.0–2.0	0.25 ± 0.86	0.0–3.0
Flip	33.67 ± 15.22	9.0–63.0	10.42 ± 8.09 ** &	1.0–25.0
Lob	21.25 ± 13.52	4.0–45.0	11.33 ± 5.92 $	1.0–21.0
Drive	24.58 ± 16.68	2.0–57.0	25.25 ± 17.33	4.0–62.0
Topspin	132.58 ± 59.48	69.0–245.0	109.42 ± 42.41	57.0–171.0
Push	64.67 ± 27.25	24.0–117.0	78.00 ± 43.35 $	29.0–185.0
Block	50.75 ± 27.54	12.0–105.0	43.08 ± 32.94	10.0–120.0
Smash	4.92 ± 5.72	0.0–21.0	8.92 ± 7.59	0.0–29.0
Chop	2.83 ± 2.88	0.0–8.0	54.50 ± 74.96 **&	0.0–234.0
Otther	5.50 ± 3.96	1.0–11.0	3.50 ± 2.68 $	0.0–8.0

** *p* < 0.01 differences male vs. female; $: medium effect size; &: large effect size; values represented in X ± SD.

**Table 5:** Tactical winning and losing game actions.

Parameters (n)	Male (n = 24)	Range	Female (n = 24)	Range
Winning game actions				
Forehand	14.50 ± 6.50	6.0–27.0	9.83 ± 2.85 + &	5.0–16.0
Backhand	7.08 ± 3.63 * &	0.0–13.0	4.42 ± 1.83 + * ^µ^ &	1.0–8.0
Losing game actions				
Forehand	40.33 ± 11.72	22.0–58.0	33.75 ± 8.18 ^µ^	24.0–56.0
Backhand	37.50 ± 12.02	22.0–58.0	32.08 ± 13.95	17.0–59.0

^µ^: Non-parametric Mann–Whitney U-test; + *p* < 0.05 differences male vs. female; * *p* < 0.05 differences forehand vs. backhand; &: large effect size; values represented in X ± SD.

**Table 6:** Winning technical–tactical actions.

Parameters (n)	Male (n = 24)	Range	Female (n = 24)	Range
Topspin F	9.25 ± 5.06 +	3.0–17.0	4.92 ± 2.46 * + ^µ^ $	1.0–0.0
Topspin B	2.83 ± 1.94	0.0–6.0	0.42 ± 0.66 ** ^µ^ &	0.0–2.0
Push F	0.67 ± 0.88	0.0–3.0	0.50 ± 0.79 ^µ^	0.0–2.0
Push B	0.67 ± 1.15	0.0–4.0	0.50 ± 0.67 ^µ^	0.0–2.0
Flip F	1.17 ± 1.26	0.0–4.0	0.58 ± 0.99 ^µ^	0.0–3.0
Flip B	0.92 ± 0.90	0.0–2.0	0.08 ± 0.28 * ^µ^ $	0.0–1.0
Block F	0.92 ± 1.50	0.0–4.0	0.75 ± 0.96 ^µ^	0.0–4.0
Block B	1.83 ± 1.33	0.0–4.0	1.58 ± 1.37	0.0–4.0
Service F	0.92 ± 0.99	0.0–3.0	0.33 ± 0.65 ^µ^	0.0–2.0
Service B	0.00 ± 0.00	0.0–0.0	0.08 ± 0.28	0.0–1.0
Lob F	0.33 ± 0.65	0.0–2.0	0.00 ± 0.00	0.0–0.0
Lob B	0.08 ± 0.28	0.0–1.0	0.00 ± 0.00	0.0–0.0
Drop F	0.08 ± 0.28	0.0–2.0	0.25 ± 0.8 ^µ^	0.0–3.0
Chop F	0.00 ± 0.00	0.0–0.0	0.25 ± 0.62	0.0–2.0
Chop B	0.00 ± 0.00	0.0–0.0	0.17 ± 0.38	0.0–1.0
Smash F	0.67 ± 0.98	0.0–2.0	1.75 ± 1.71 ^µ^	0.0–6.0
Smash B	0.00 ± 0.00	0.0–0.0	0.08 ± 0.28	0.0–1.0
Drive F	0.25 ± 0.45	0.0–1.0	0.50 ± 0.67 ^µ^	0.0–2.0
Drive B	0.75 ± 0.96	0.0–3.0	1.42 ± 1.50 ^µ^	0.0–4.0

^µ^: Non-parametric Mann–Whitney U-test; * *p* < 0.05; ** *p* < 0.01; differences male vs. female; + *p* < 0.05 differences F vs. B; F: forehand; B: backhand; $: medium effect size; &: large effect size, values represented in X ± SD.

**Table 7:** Losing technical–tactical actions.

Parameters	Male (n = 24)	Range	Female (n = 24)	Range
Topspin F	19.75 ± 9.01	8.0–34.0	16.50 ± 5.85	10.0–25.0
Topspin B	8.58 ± 4.48 +	2.0–17.0	7.08 ± 5.26 + ^µ^	0.0–19.0
Push F	3.75 ± 2.37	0.0–7.0	2.42 ± 1.62 * ^µ^ $	0.0–5.0
Push B	2.17 ± 1.58	0.0–4.0	2.42 ± 2.15 ^µ^	0.0–7.0
Flip F	4.00 ± 2.37	1.0–8.0	1.75 ± 2.17 *	0.0–8.0
Flip B	3.08 ± 1.97	1.0–6.0	0.83 ± 1.11 * ^µ^ &	0.0–3.0
Block F	3.00 ± 2.69	0.0–8.0	3.75 ± 3.49 ^µ^	1.0–12.0
Block B	12.67 ± 7.57 +	3.0–28.0	10.58 ± 7.36 + ^µ^ $	2.0–25.0
Service F	1.50 ± 1.44	0.0–4.0	0.67 ± 0.77	0.0–2.0
Lob F	2.67 ± 2.06	0.0–7.0	1.92 ± 1.73 ^µ^	0.0–5.0
Lob B	2.92 ± 2.10	0.0–6.0	1.50 ± 1.31 ^µ^	0.0–4.0
Chop F	0.17 ± 0.38	0.0–1.0	1.33 ± 2.34 ^µ^	0.0–7.0
Chop B	0.67 ± 0.98	0.0–1.0	4.17 ± 4.76 ^µ^	0.0–11.0
Smash F	0.50 ± 0.67	0.0–2.0	1.75 ± 3.01 ^µ^	0.0–11.0
Drive F	1.25 ± 1.71	0.0–6.0	0.83 ± 1.19 ^µ^	0.0–4.0
Drive B	5.75 ± 4.02 +	0.0–12.0	4.17 ± 3.83 +	0.0–12.0

^µ^: Non-parametric Mann–Whitney U-test; * *p* < 0.05; differences male vs. female; + *p* < 0.05 differences D vs. R; R: forehand; B: backhand; $: medium effect size; &: large effect size; values represented in X ± SD.

**Table 8:** Direction and pivot footwork game.

Parameters (n)	Male (n = 24)	Range	Female (n = 24)	Range
Diagonal	141.17 ± 60.78	62.0–236.0	156.17 ± 83.89 ^µ^	75.0–357.0
Parallel	122.83 ± 43.65	50.0–196.0	124.83 ± 57.22	49.0–249.0
Pivot footwork	58.75 ± 34.04	32.0–134.0	31.67 ± 27.02 *$	4.0–102.0

^µ^: Non-parametric Mann–Whitney U-test; * *p* < 0.05; differences male vs. female; $: medium effect size; values represented in X ± SD.

## Data Availability

Data are expressed in the present study.
